# The effects of exercise training on hypothalamic-pituitary-adrenal axis reactivity and autonomic response to acute stress—a randomized controlled study

**DOI:** 10.1186/s13063-020-04803-3

**Published:** 2020-10-27

**Authors:** Elin Arvidson, Anna Sjörs Dahlman, Mats Börjesson, Lennart Gullstrand, Ingibjörg H. Jonsdottir

**Affiliations:** 1 The Institute of Stress Medicine, Västra götalandsregionen, Gothenburg, Sweden; 2 Swedish National Road and Transport Research Institute, Gothenburg, Sweden; 3 The Department of Food and Nutrition, and Sport Science, Faculty of Education, University of Gothenburg, Gothenburg, Sweden; 4 The Department of Neuroscience and Physiology, Gothenburg University and Sahlgrenska University Hospital/Östra Gothenburg, Gothenburg, Sweden

**Keywords:** Physical activity, Longitudinal study, Psychosocial stress

## Abstract

**Background:**

Exercise training is suggested to have a stress-buffering effect on physiological reactions to acute stress. The so-called cross-stressor adaptation hypothesis is one of many theories behind the plausible effects, proposing that the attenuated physiological reaction seen in trained individuals in response to acute exercise is also seen when the individual is exposed to acute psychosocial stress. However, few randomized controlled trials (RCT) are available in this field. Therefore, the aim of the present trial was to study the effects of a 6-month aerobic exercise intervention on the physiological response to acute laboratory stress.

**Methods:**

A two-armed RCT including untrained but healthy individuals aged 20–50 years was conducted. Assessments included a peak oxygen uptake test and a psychosocial stress test (the Trier Social Stress Test). A total of 88 participants went through both baseline and follow-up measures (48 in the intervention group and 40 in the control group) with a similar proportion of women and men (20 women and 28 men in the intervention group and 18 women and 22 men in the control group). Outcome measures were adrenocorticotrophic hormone, cortisol, systolic and diastolic blood pressure, and heart rate responses to acute psychosocial stress.

**Results:**

Oxygen uptake and time-to-exhaustion increased significantly following the intervention, while a decrease was seen in the control group. The analyses showed attenuated responses to acute psychosocial stress for all variables in both groups at follow-up, with no differences between the groups. No correlation was seen between amount of exercise training and reactivity to the stress test. Despite the increased oxygen uptake in the intervention group, no differences were seen between the groups for any of the outcome variables at follow-up.

**Conclusions:**

In this study, the cross-stressor adaptation hypothesis could not be confirmed. Both groups showed decreased reactions indicating a habituation to the stress test.

**Trial registration:**

ClinicalTrials.gov NCT02051127. Registered on 31 January 2014—retrospectively registered.

## Background

Regular exercise training has been shown to play a great role for health, not only for somatic complaints, cardiovascular and overall mortality, but also for mental well-being [1–4]. Also, exercise training has been proposed as a buffer for the detrimental effects of stress [5]. The mechanisms behind the possible stress-buffering effects of exercise and fitness are still not fully known but seem to be related to both physiological and psychological aspects. One of many plausible mechanisms is the so-called cross-stressor adaptation hypothesis, described by Sothman et al. in 1996. The theory suggested that the attenuated physiological reaction seen in trained individuals in response to acute exercise is also valid in response to acute psychosocial stress [6]. However, previous research is not consistent regarding the effect of exercise training on physiological stress responses.

The central physiological stress response systems are the hypothalamic-pituitary-adrenal (HPA) axis and the autonomic nervous system [7, 8]. When exposed to acute stress, the HPA axis is activated resulting in increased levels of the stress hormones adrenocorticotropic hormone (ACTH) and cortisol. A similar pattern is seen for the autonomic responses, where the onset of stress will increase heart rate and blood pressure. These responses are usually adequate and essential for the body to be able to overcome increased metabolic demands. In everyday life with recurrent onsets of stress, the magnitude of the physiological response may play a role in health. Thus, if the stress reaction is frequently triggered without sufficient time to recover, it could result in elevated basal levels of stress hormones, blood pressure, and heart rate with the risk of deteriorating health as a consequence [9–11].

An early review concluded that aerobically fit individuals exhibited a lower grade of autonomic stress response (e.g., heart rate, blood pressure, or subjective experience of the task) compared to unfit individuals [12]. Later, van Doornen et al. [13] suggested that it is not possible to predict an individual’s autonomic response to psychosocial stress by the level of fitness, partly because of the varying definitions of fitness, and partly because of the different mechanisms involved in physical and psychosocial stress. This was later confirmed by Jackson and Dishman [14], finding that when fitness was measured as peak oxygen uptake (VO_2_ peak), the effect was smaller than when self-reported levels of exercise were used. It might depend on the genetic component in fitness, which seems to be of importance for an individual’s ability to increase VO_2_ peak [15]. In the same year, Forcier et al. [16] published a paper showing that fitness was related to a less pronounced reaction of heart rate and systolic blood pressure in response to acute psychosocial stress. However, the definition of fitness was not specified and only a few of the longitudinal studies included in the analysis involved a control group. Thus, agreement has not yet been reached regarding whether the cross-stressor adaptation hypothesis applies to autonomic reactions.

The relationship between HPA axis response and exercise training in connection to acute psychosocial stress has not been as thoroughly studied as the autonomic reactions. Moreover, available studies show different results, also regarding sex differences. Klaperski [17] found that trained young women (18–28 years) showed a less pronounced physiological response, measured as salivary cortisol, to an acute psychosocial stress test compared to untrained women. Likewise, Rimmele et al. [18] showed that male elite athletes showed a lower reaction for cortisol, blood pressure, and heart rate compared to untrained men in response to acute psychosocial stress. This was recently confirmed by Gerber et al. [19], presenting similar results in young male and female students. Thus, participants reporting high self-reported perceived stress and low level of physical activity showed a more pronounced increase in cortisol in response to psychosocial stress test than participants reporting a low level of stress and high level of physical activity. No sex differences were seen in this study. In contrast, Childs and de Wit [20] did not support these results, showing no effect of regular exercise on saliva cortisol response to acute psychosocial stress. Furthermore, in this study, men responded with a greater cortisol response to the stressor than women. In conformity with the results from Childs and de Wit, a recent cross-sectional study found no associations between cardiorespiratory fitness, cortisol, blood pressure, and heart rate in women, in response to an acute psychosocial stress test [21].

Most of the above-mentioned studies were cross-sectional. Longitudinal studies are few, and only one randomized controlled trial (RCT) was found measuring HPA-axis response to acute psychosocial stress in relation to exercise. Klaperski et al. [22] examined the effect of exercise training on the HPA-axis reactivity in untrained individuals, compared to a control group. The group that performed aerobic exercise showed a reduced reactivity to acute stress regarding levels of cortisol, heart rate, and heart rate variability compared to the control group. However, a reference group performing relaxation training also showed a reduced cortisol reaction compared to the control group, which did not allow firm conclusions whether the effects seen are solely due to exercise training. Thus, there is a need to further study the effects of exercise on physiological responses to acute stress.

Therefore, the primary aim of this RCT was to examine the effects of a 6-month aerobic exercise training intervention on HPA axis reactivity to acute psychosocial stress in untrained individuals. As the secondary aim, we also studied the effects on autonomic responses. The hypothesis is that aerobic exercise training will result in attenuated HPA axis reactivity, assessed by ACTH and cortisol levels, and an attenuated autonomic response, assessed as blood pressure and heart rate, in response to acute psychosocial laboratory stress.

## Methods

This study was a two-armed RCT, designed according to the Consolidated Standards of Reporting Trials (CONSORT) Statement [23] (checklist is available as an additional file), and registered at ClinicalTrials.gov (ID NCT02051127). Only the primary outcome measures from the original study protocol will be presented in this paper. Baseline evaluations included a VO_2_ peak test and a psychosocial stress test (see descriptions below). After initial tests, the participants were randomized to either the control or the intervention group. The randomization was done by a nurse, using sealed envelopes and a 50/50% distribution to the intervention and control groups. Since the adherence to the intervention protocol was lower than expected in the intervention group, the distribution of randomization was changed to 70% for the intervention group and 30% for the control group during the last year of inclusion.

The intervention group performed aerobic exercise training for 6 months, while the control group was asked to maintain their current level of physical activity. Both groups then underwent the same tests as were conducted at baseline. The enrollment started at spring in 2013 and lasted to spring in 2016, with the last follow-up performed in the autumn of 2016. The testing of participants was performed once per month all year, with approximately eight to twelve individuals per time, to avoid seasonal effects. The method has previously been described in detail elsewhere [24].

### Participants

Inclusion criteria were as follows: age 20–50 years, essentially healthy (not suffering from any known somatic or psychiatric disease such as diabetes, heart disease, or stress-related diseases), and working or studying at least 50% of full time. In addition, the individuals should not have performed any regular exercise during the last year and rate themselves as being mostly sedentary, corresponding to level 1 in the Saltin Grimby Activity 4-Level Scale [25, 26]. Exclusion criteria were as follows: glucose level ≥ 7 mmol/L; HbA1C ≥ 48 mmol/mol; diverging electrocardiography; blood pressure above 140/90 mmHg; anemia (Hb < 120 g/L for women, < 130 g/L for men); body mass index less than 18.5 or above 35 kg/m^2^; medication with beta blockers, psychopharmacological drugs, or asthmatic medicine; and inability to exercise at a relatively high intensity.

**Fig. 1:**
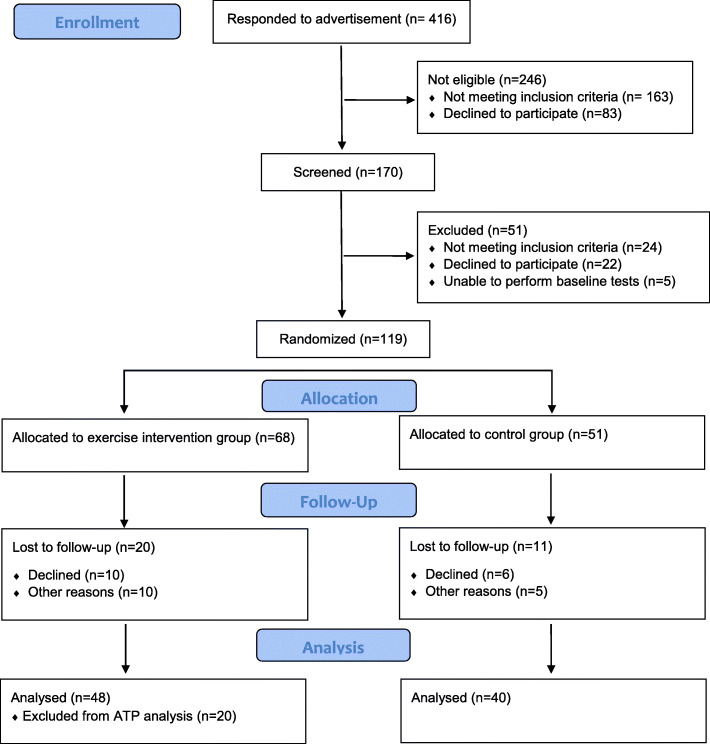
Study flow diagram. ATP, adherence to protocol

Information about the study was distributed by advertisement in two major newspapers in the district around Gothenburg in western Sweden and through notice boards and social media. The number of responders to the advertisement for the study was 416. Of these, 170 individuals were eligible according to the inclusion criteria and were offered a physical screening to test for exclusion criteria, resulting in 24 individuals being excluded. A further 22 individuals declined participation or did not enter the study for other reasons (Fig. 1). A final number of 119 individuals went through the baseline tests.

### Peak oxygen uptake test

To assess the aerobic capacity and heart rate of the participants, a VO_2_ peak test was conducted at the Centre for Health and Performance, University of Gothenburg. The test was performed on a bicycle ergometer (Monark, 828 E, Monark Exercise AB, Vansbro, Sweden). The participants started the test by warming up for 5 min with a cadence of 70 rpm at a low resistance (50 W). The VO_2_ peak test was a ramp test with increasing resistance and the same cadence as during warm up. The women started at 87.5 W, increasing by 17.5 watts every minute until exhaustion. The men started at 105 W, also increasing by 17.5 W until exhaustion (VO_2_ leveling off or respiratory exchange ratio > 1.1 and inability to keep the cadence). A total work of 5–8 min (time-to-exhaustion) was considered as optimal for reaching peak values. Peak oxygen uptake, expressed as mL/kg/min, was measured with the Jaeger Oxycon Pro metabolic chart (Carefusion, Hoechberg, Germany) in the mixing chamber mode. The device was calibrated before each measurement according to the manufacturer’s manual. During the test, the heart rate (beats per minute, bpm) was monitored and measured with a pulse sensor (Polar 300 RS, Polar, Finland).

### Psychosocial stress test

One week after the oxygen uptake test, the Trier Social Stress Test (TSST) was performed at the Institute of Stress Medicine in Gothenburg between 1 p.m. and 3 p.m. The TSST is the most commonly used laboratory stress test developed to elicit a physiological reaction to acute psychosocial stress in a standardized setting [27]. The TSST has been shown to have good validity and reliability and is widely used in this research area [20, 28, 29]. Before arriving at the lab, the participants had ingested a standardized meal containing controlled amounts of fat, protein, and carbohydrates, approximately 2 h before the test. After a short rest, the participant entered the test room and was given instructions in front of a committee consisting of three members. A video camera and a microphone were installed, and they were falsely told that they were video and audio taped for later analyses of their behavior and voice. After 5 min of preparation in another room, the participant re-entered the test room. The first part of the test was a 5-min free speech, where the participants were asked to apply for their dream job in front of the committee. The topic was slightly modified in the follow-up session, where the participant instead was asked to apply for a job they dreamed about as a child. The committee gave no form of response to the participant during the speech. The second part was an arithmetic task (serial subtraction), also 5 min long, and after the test, the participant left the room. To study the recovery after the test, the participants rested for 60 min in a calm setting.

### Exercise training intervention

One week after randomization, participants allocated to the intervention group were called for a group meeting where they received information about the intervention and were instructed to start regular aerobic exercise training. Aerobic exercise is the most studied form of exercise in this field and was chosen to enable comparisons with other studies. The duration of 6 months was set to enable a gradual increase of their level of activity and to have sufficient time to reach physiological changes. The goal was to reach an exercise level of three times per week, 45–60 min per session and with an average heart rate of at least 75% of peak heart rate, measured at the VO_2_ peak test. To monitor the intensity and duration of exercise, the participants used a heart rate monitor (Garmin Forerunner® 210, USA), at every training session and transferred the data recorded directly to an internet-based training log (www.funbeat.se). The participants were free to choose what type of activity to perform, and if they wanted to exercise individually or together with others, as long as the goal for the average heart rate was fulfilled. The participants were asked to avoid performing resistance training during the intervention period, since resistance training might affect the outcome variables in a different way than aerobic exercise [30]. They were also offered four meetings with a trained coach to identify strengths and potential obstacles to getting through the intervention. The participants were given 1-year free access to a commercial fitness establishment (Nordic Wellness) with several facilities in and around Gothenburg to further facilitate adherence to training.

Individuals in the control group were instructed to keep their activity level unchanged during the 6-month intervention period and refrain from any exercise training. After the follow-up, they were encouraged to start exercising and received 1-year free access to the same fitness establishment as the intervention group.

### Outcome measurements

The primary outcome of the study was HPA-axis response to acute psychosocial stress, assessed as plasma ACTH and total serum cortisol response, before and after the intervention. The participants were provided with a peripheral venous catheter in an antecubital vein (BD Venflon Pro, Becton Dickinson Infusion Therapy, USA), and a total of seven blood samples were drawn during each TSST. An initial sample was drawn 10 min before the test started (− 10). The second was drawn immediately before the test (− 0), and the third immediately after the test was finished (+ 0). Remaining samplings were made 10, 20, 40, and 60 min after the test in order to study the response and the recovery. Samples for serum were collected in EDTA tubes, and samples for plasma were collected in Serum Sep Cloth Activator tubes. To separate the plasma, tubes were cold spun at 3500 rpm for 15 min and stored at − 80 °C until analyzed. To separate serum, tubes were spun at 20 °C for 10 min at 3500 rpm and stored at 4 °C until analyzed the day after the test. Plasma concentrations of ACTH were assessed by immunoradiometric assay (limit of detection, 0.4 pmol/L) (CIS bio International, Gif-sur-Yvette Cedex, France). Serum concentrations of cortisol were assessed by electro chemiluminescence immunoassay (limit of detection, 0.5 nmol/L) (Roche Diagnostics GmbH, Mannheim, Germany). To assess the secondary outcomes, autonomic reactions in terms of heart rate, and systolic and diastolic blood pressure, an automatic blood pressure cuff (Welch Allyn, ABPM 6100, USA) was used. The device was assessing every 5 min from 10 min before the TSST to 60 min after the test.

### Data handling

Pre-test values were the mean value of the − 10 and − 0 values taken before the test started, peak value was the highest value after the test (10 or 20 min for ACTH and cortisol and during or directly after for systolic and diastolic blood pressure and heart rate). For ACTH and cortisol, the lowest value was the last one (60 min) for all participants. For systolic and diastolic blood pressure and heart rate, that are more responsive, the lowest value occurred between 10 and 60 min after the test.

The *reactivity* was defined as percental change from pre-test to peak value and was calculated by dividing the absolute change with the pre-test value. *Recovery* was defined as percental change from peak to lowest value and was calculated by dividing the absolute change with the peak value. Non-responders in HPA axis were defined as a zero- or negative response in ACTH and/or cortisol from pre-test to peak values.

### Statistics

A sample size calculation for the main outcome measure cortisol showed that 39 subjects in each group were needed to be able to detect an effect size Cohen’s *f* = .25, with power ≥ .80 and a two-sided *α* = .05 (G*power 3.1). Expecting that several of dropouts would occur, the goal was to include 50 subjects in each group.

To analyze group differences in the physiological response to TSST at baseline and follow-up, mixed between-within subjects analysis of variance (ANOVA) were used for ACTH, cortisol, systolic and diastolic blood pressure, and heart rate at baseline and follow-up using all seven time points in the analysis.

To study the effects of the intervention, group differences, and changes from baseline to follow-up for the three points pre-test, peak and lowest value after the test were analyzed with mixed between-within subjects’ ANOVA.

In the third step, three summarizing measures were performed. To study the total output of ACTH and cortisol, area under the curve with respect to increase (AUC_i_) [31] was calculated from pre-test to 60 min after the stress test. As a measure of reactivity to and recovery from the psychosocial stress test, percental response from pre-test to peak and percental recovery from peak to lowest value was used for all outcome measures. Mixed between-within subjects ANOVA was performed to study differences from baseline to follow-up for these parameters.

We were also interested in whether the number of training sessions during the intervention correlated with the response in ACTH, cortisol, systolic and diastolic blood pressure, and heart rate. For this, Pearson’s correlation analyses were used.

Since women and men were analyzed together, adjustments were made for sex in all analyses mentioned above. If the adjusted result did not change from the crude analysis, the results from the unadjusted analyses are displayed in the tables. All participants included in the study who performed both baseline and follow-up measures were included in the initial analysis according to the intention-to-treat principle. All analyses were re-performed after exclusion of outliers, which was defined as values higher or lower than two standard deviations from the mean.

The lowest accepted level of exercise was set to at least two times per week during at least half of the intervention period (a minimum of 26 sessions). In the first sub-analysis, only participants who had reached the accepted level of training sessions were included, according to the adherence-to-protocol principle. In the second sub-analysis, participants defined as non-responders in ACTH and cortisol from pre-test to peak value at baseline were excluded.

All data were analyzed with IBM SPSS Statistics for Windows, Version 22.0. Armonk, NY, USA. For normally distributed data, values are presented as means and standard deviations, with a significance level set to *p* <  0.05. For not normally distributed data, results are presented as geometric mean and anti-logged confidence intervals (CI). Effect size is presented as eta squared. Original data presented in this paper are available on request.

## Results

The number of individuals randomized to either the intervention or the control group was 119, with 68 participants randomized to the intervention group and 51 to the control group. The dropout rate was 20 individuals in the intervention group and 11 in the control group due to unwillingness to go through retesting (*n* = 16), injuries (*n* = 4), changed working conditions (*n* = 4), starting anti-depressant medication (*n* = 4), pregnancy (*n* = 2), and randomization to the control group but started to exercise (*n* = 1). In total, 88 participants (72%) (48 in the intervention group and 40 in the control group) went through both baseline and follow-up measures (*see study flow diagram*, Fig. 1). There was an even distribution of women and men in both groups, with 18 women and 22 men in the control group and 20 women and 28 men in the intervention group. For baseline characteristics of the participants, see Table 1. Due to missing samples for some individuals at different time points, the number of included individuals (*n*) varies between different outcomes.

**Table 1:** Baseline characteristics of the participants

	Control		Intervention	
	Mean	SD	Mean	SD
Sex (women/men)	18/22		20/28	
Age (range)	41 (24–50)	7.8	38 (23–49)	6.7
BMI	24.7	3.9	25.0	3.1
WHR	0.9	0.1	0.9	0.1
Post-secondary education	85%		92%	
Tobacco user	20%		13%	
VO_2_ peak (mL/kg/min)	33.4	6.2	34.8	6.8
TTE (min:sec)	7:19	02:32	7:58	02:23

*BMI* body mass index, *WHR* waist-to-hip ratio, *VO*_*2*_ *peak* peak oxygen uptake, *TTE* time-to-exhaustion, *SD* standard deviation

### Adherence to protocol

The mean number of training sessions in the intervention group was 33 sessions per person (range 0 to 77) out of a possible maximum of 78 sessions during the intervention period. After exclusion of participants who performed fewer than 26 sessions, the mean number of sessions for the remaining participants was 45 (range 27 to 77). Thus, in the adherence-to-protocol analysis, 20 subjects in the intervention group who did not reach the intended level of physical exercise were excluded. Participants excluded from the adherence-to-protocol analysis did not significantly differ from participants following the protocol regarding sex, age, BMI, education, tobacco use, VO_2_ peak, or time-to-exhaustion.

### Oxygen uptake

In the age group 20–29 years, the mean oxygen uptake at baseline was 37.5 mL/kg^/^min for both women and men (*n* = 6 and *n* = 5, respectively). In the age groups 30–39 and 40–50 years, the mean values for women were 30.1 and 29.0 mL/kg/min, respectively (*n* = 11 and *n* = 21), and for men 37.7 and 36.3 mL/kg^/^min, respectively (*n* = 21 and *n* = 24). At follow-up, the VO_2_ peak and time-to-exhaustion had increased significantly in the intervention group (9.4% and 11.0%, respectively) between baseline and the 6-month follow-up (both measures *p* <  0.001*)*. When including only participants following the intervention protocol, the increase in VO_2_ peak was 9.4%, and the increase in time-to-exhaustion was 9.5%. At the same time, the control group decreased their VO_2_ peak (− 3.0%, *p* = 0.018) and time-to-exhaustion (− 0.7%, *p* = 0.713). A mixed between-within subjects ANOVA for VO_2_ peak confirmed the significant effects of time (*F* [1, 81] = 12.33, *p* = 0.001, eta squared 0.13). Also, the group differences (*F* [1, 81] = 6.74, *p* = 0.011, eta squared = 0.08) and interaction effects (*F* [1, 81] = 41.81, *p* <  0.001, eta squared = 0.34) were confirmed, showing that the intervention increased oxygen uptake. Likewise, time-to-exhaustion showed significant effects of time (*F* [1, 86] = 20.71, *p* <  0.001, eta squared 0.19), group (*F* [1, 86] = 4.11, *p* = 0.046, eta squared 0.05), and interaction (*F* [1, 86] = 26.50, *p* <  0.001, eta squared 0.24), showing that the intervention also affected the total duration of the test.

### HPA axis response to acute psychosocial stress

**Fig. 2:**
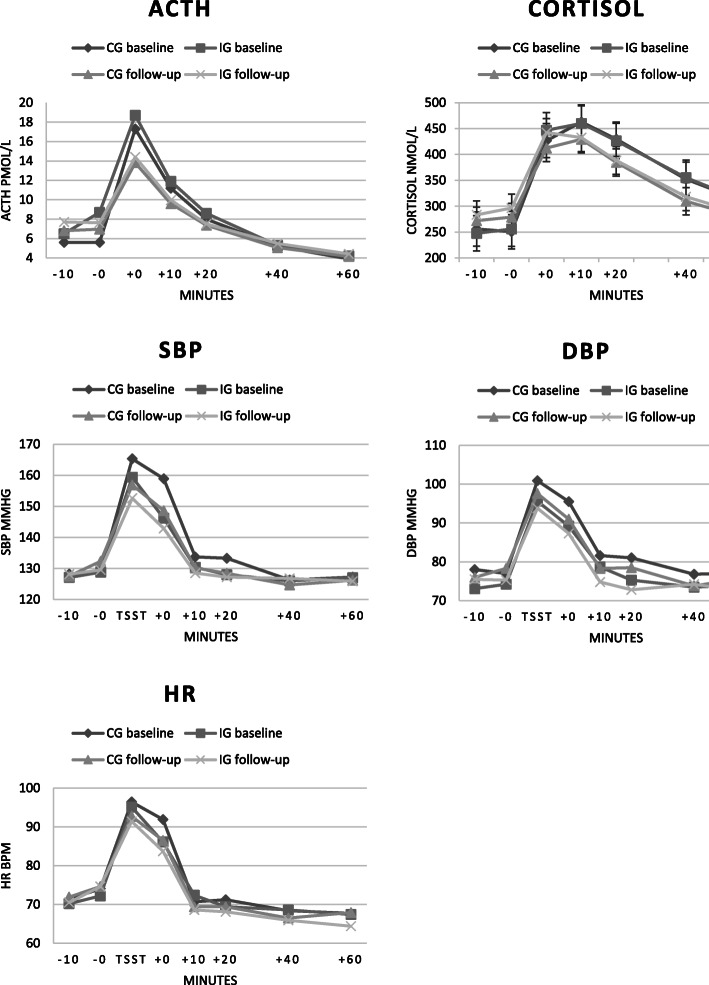
HPA-axis and autonomic responses to the psychosocial stress test

The HPA-axis responses to the TSST are shown in Fig. 2. At baseline TSST, four participants showed no ACTH response and additional four participants showed no cortisol response to acute stress.

Mean values for the seven time points are presented for cortisol, while ACTH was not normally distributed and thus geometric means are displayed. At baseline, the mixed between-within subjects ANOVA for ACTH, including all seven time points, showed a significant effect of time, and follow-up measures gave the same result (Table 2), confirming significant physiological reactions to acute stress. The exclusion of participants not following the protocol did not change the results. Also, for cortisol, baseline results showed a significant effect of time. At follow-up, the interaction effect was significant as well, with the intervention group having a greater response between time point two and three compared to the control group. However, when excluding participants not following the protocol, the interaction effect was no longer significant (*F* [6, 56] = 1.88, *p* = 0.100). The exclusion of outliers did not change the results.

**Table 2:** Mixed between-within subjects ANOVA for all seven time points at baseline and follow-up TSST

	*n*	Time			Group			Time*group		
		*F*	*p*	Eta squared	*F*	*p*	Eta squared	*F*	*p*	Eta squared
**Baseline**										
ACTH (pmol/L)*	82	4.94	**< 0.001**	0.29	1.77	0.118	0.13	2.07	0.154	0.03
Cortisol (nmol/L)*	79	7.01	**< 0.001**	0.37	0.04	0.850	0.00	1.25	0.291	0.10
SBP (mmHg)*	64	4.81	**0.001**	0.34	3.33	0.073	0.05	3.66	**0.004**	0.28
DBP (mmHg)*	66	4.91	**< 0.001**	0.34	5.01	**0.029**	0.07	0.70	0.649	0.07
HR (Bpm)	66	17.20	**< 0.001**	0.64	0.00	0.962	0.00	2.28	**0.048**	0.19
**Follow-up**										
ACTH (pmol/L)*	86	6.64	**< 0.001**	0.34	2.23	0.139	0.03	1.03	0.410	0.07
Cortisol (nmol/L)*	84	8.10	**< 0.001**	0.39	0.20	0.655	0.00	2.25	**0.048**	0.15
SBP (mmHg)*	70	3.22	**0.008**	0.24	0.00	0.999	0.00	0.82	0.556	0.07
DBP (mmHg)*	69	5.27	**< 0.001**	0.34	1.24	0.270	0.02	1.82	0.110	0.15
HR (Bpm)	70	19.84	**< 0.001**	0.65	1.45	0.233	0.02	1.41	0.225	0.12

*ACTH* adrenocorticotropic hormone, *SBP* systolic blood pressure, *DBT* diastolic blood pressure, *HR* heart rate, *Bpm* beats per minute

*Adjusted for sex

Values and results from the mixed between-within subjects ANOVA for the physiological reactivity to TSST are shown in Table 3. Significant effects of time were seen for ACTH and cortisol, showing decreased responses at the follow-up TSST compared to the baseline TSST. No effects of group and no interaction effects were seen for neither ACTH nor cortisol. Mixed between-within subjects ANOVA on recovery from the psychosocial stress test showed no significant differences between the groups for ACTH, but when excluding outliers a significant result was seen for time (*F* [1, 83] = 15.9, *p* <  0.001, eta squared = 0.161). For cortisol, there was a significant effect of time showing an increased recovery at follow-up compared to baseline.

**Table 3:** Values for percental reactivity to and recovery from the psychosocial stress test, along with results from the mixed between-within subjects ANOVA for reactivity and recovery at baseline and follow-up

	*n*	**Percental reactivity**				**Mixed between-within subjects ANOVA**								
		Control group		Intervention group		Baseline to follow-up			Group			Time*group		
		Baseline	Follow-up	Baseline	Follow-up	*F*	*p*	Eta squared	*F*	*p*	Eta squared	*F*	*p*	Eta squared
ACTH (pmol/L)	85	261 (159; 364)	108 (69; 147)	215 (157; 273)	100 (62; 139)	25.67	**< 0.001**	0.236	0.42	0.518	0.01	0.35	0.555	0.00
Cort (nmol/L)	81	96 (73; 119)	73 (53; 94)	94 (79; 109)	60 (51; 70)	17.76	**< 0.001**	0.18	0.64	0.426	0.01	0.60	0.442	0.01
SBP (mmHg)*	83	34 (29;39)	24 (21; 28)	29 (25; 34)	23 (19; 27)	0.85	0.360	0.01	2.68	0.106	0.03	1.00	0.321	0.01
DBP (mmHg)	83	26 (31; 40)	30 (26; 34)	36 (30; 43)	29 (25; 33)	7.61	**0.007**	0.09	0.00	0.964	0.00	0.12	0.727	0.00
HR (Bpm)*	78	44 (35; 53)	20 (12; 28)	40 (33; 47)	25 (18; 33)	0.04	0.846	0.00	0.08	0.778	0.00	2.15	0.147	0.03
	*n*	**Percental recovery**				**Mixed between-within subjects ANOVA**								
		Control group		Intervention group		Baseline to follow-up			Group			Time*group		
		Baseline	Follow-up	Baseline	Follow-up	*F*	*p*	Eta squared	*F*	*p*	Eta squared	*F*	*p*	Eta squared
ACTH (pmol/L)*	87	68 (62; 74)	60 (53; 67)	70 (64; 75)	65 (61; 69)	0.94	0.334	0.01	1.17	0.282	0.01	1.03	0.313	0.01
Cort (nmol/L)	84	33 (29; 37)	38 (35; 41)	36 (33; 39)	39 (37; 41)	7.87	**0.006**	0.09	0.60	0.442	0.01	0.41	0.526	0.01
SBP (mmHg)*	85	29 (27; 31)	27 (24; 29)	27 (26; 29)	25 (22; 27)	0.55	0.459	0.01	2.59	0.112	0.03	0.00	0.99	0.00
DBP (mmHg)	85	31 (28; 33)	31 (28; 34)	31 (29; 34)	31 (29; 34)	0.28	0.596	0.00	0.18	0.674	0.00	0.13	0.717	0.00
HR (Bpm)*	84	30 (26; 35)	33 (29; 36)	36 (32; 40)	36 (33; 38)	0.35	0.557	0.00	3.12	0.081	0.04	1.68	0.199	0.02

*Adjusted for sex

*ACTH* adrenocorticotropic hormone, *SBP* systolic blood pressure, *DBT* diastolic blood pressure, *HR* heart rate, *Bpm* beats per minute

### Effects of exercise training on HPA-axis AUC_i_

The change in ACTH AUC_i_ from baseline to follow-up was significant, showing decreased values for both groups at follow-up (*F* [1, 80] = 10.76, *p* = 0.002, eta squared = 0.119). No significant group or interaction effects were seen (*F* [1, 80] = 1.32, *p* = 0.255 and *F* [1, 80] = 0.51, *p* = 0.477, respectively). The same result was shown for cortisol AUC_i_, with a significant effect of time (*F* [1, 75] = 41.57, *p* <  0.001, eta squared = 0.357) but no group or interaction effects (*F* [1, 75] = 0.00, *p* = 0.992 and *F* [1, 75] = 0.16, *p* = 0.688, respectively). The exclusion of outliers did not change the results.

### Autonomic response to acute psychosocial stress

Response curves with mean values for the seven time points for systolic and diastolic blood pressure and heart rate are shown in Fig. 2, and the results from the mixed between-within subjects ANOVA are displayed in Table 2. At baseline, results for systolic blood pressure showed significant effects of time and interaction, with both groups showing reactions to the stress test, and the intervention group having a greater response than the control group. At follow-up, only time was significant, confirming a response to the stress test. For diastolic blood pressure, the baseline results showed significant effects of time and group, showing reactions to the stress test in both groups and the control group having higher values than the intervention group. At follow-up, only time was significant. For baseline heart rate, significant effects were seen of time and interaction, with both groups showing a response to the stress test, and the control group having a smaller recovery. The follow-up results did only show significant effects of time. Excluding participants not reaching the goal for exercise training did not change these results.

Values and results from the mixed between-within subjects ANOVA for the physiological reactivity to TSST are shown in Table 3. Significant effects of time were seen for diastolic blood pressure, showing a decreased reactivity at follow-up compared to baseline, but not for systolic blood pressure or heart rate. When excluding outliers, a significant effect of time was seen also for systolic blood pressure and heart rate (*F* [1, 77] = 24.7, *p* < 0.001, eta squared = 0.243 and *F* [1, 75] = 0.16, *p* < 0.001, eta squared = 0.376, respectively), representing a lower reactivity at follow-up compared to baseline. The analyses showed no effects of group and no interaction effects for any of the variables. For recovery from psychosocial stress, no significant effects were seen from baseline to follow-up for systolic and diastolic blood pressure and heart rate, and no differences were shown between the groups. The exclusion of outliers resulted in significant effects of time for systolic blood pressure and heart rate (*F* [1, 82] = 8.3, *p* = 0.005, eta squared = 0.092 and *F* [1, 80] = 11.7, *p* = 0.001, eta squared = 0.128, respectively), with both groups showing a decreased recovery for systolic blood pressure and an increased recovery for heart rate.

### Effects of exercise training on pre-test, peak, and lowest value

Mean values for the time points pre-test, peak, and lowest value are presented in Table 4. Variables marked with a * is presented as geometric means and its anti-logged 95% CI. Results from the mixed between-within subjects ANOVA for pre-test, peak, and lowest values from baseline to follow-up are presented in Table 5. The pre-test values were significantly higher for cortisol and heart rate at the follow-up TSST compared to the baseline TSST. When excluding outliers, this was seen also for ACTH (*F* [1, 78] = 30.9, *p* < 0.001, eta squared = 0.283). At the same time, peak values for systolic and diastolic blood pressure were lower at follow-up compared to baseline, with low effect sizes. No differences were seen for lowest value, and the exclusion of non-compliers did not change the result.

**Table 4:** Values for pre-test, peak, and lowest value at the psychosocial stress test at baseline and follow-up

	Control group			Intervention group		
	*n*	Baseline	Follow-up	*n*	Baseline	Follow-up
**Pre-test**						
ACTH*, pmol/L	40	4.6 (3.8; 5.6)	5.8 (4.8; 7.0)	45	6.1 (5.1; 7.2)	7.0 (6.2; 7.9)
Cortisol*, nmol/L	38	233 (204; 265)	254 (221; 293)	44	242 (224; 262)	277 (255; 305)
SBP, mmHg	40	129 (123; 134)	130 (124; 135)	46	128 (123; 133)	129 (125; 133)
DBP, mmHg	40	78 (74; 81)	77 (74; 81)	46	74 (70; 77)	75 (73; 78)
HR, bpm	38	73 (70; 76)	79 (76; 83)	44	71 (68; 74)	78 (76; 80)
**Peak**						
ACTH*, pmol/L	40	13.0 (10.2; 16.8)	10.6 (8.4; 13.5)	47	15.6 (13.0; 18.7)	12.6 (10.8; 14.7)
Cortisol, nmol/L	40	450 (406; 493)	439 (398; 480)	45	468 (436; 501)	456 (426; 486)
SBP, mmHg	39	171 (162; 180)	163 (156; 170)	46	164 (158; 171)	158 (151; 164)
DBP, mmHg	39	104 (100; 109)	101 (97; 105)	46	99 (95; 102)	97 (94; 100)
HR*, bpm	38	101 (95; 108)	94 (87; 99)	46	97 (92; 104)	95 (90; 99)
**Lowest value**						
ACTH*, pmol/L	40	3.5 (3.0; 4.0)	3.7 (3.2; 4.3)	47	3.9 (3.5; 4.4)	4.1 (3.6; 4.6)
Cortisol, nmol/L	40	301 (267; 334)	271 (241; 301)	45	302 (272; 331)	278 (256; 298)
SBP, mmHg	36	120 (115; 125)	118 (113; 123)	44	118 (115; 122)	118 (114; 122)
DBP, mmHg	36	72 (68; 75)	67 (65; 72)	44	68 (65; 70)	66 (64; 69)
HR, bpm	36	64 (61; 67)	63 (60; 65)	44	63 (60; 65)	61 (58; 63)

*Geometric mean and anti-logged 95% confidence intervals (CI)

*ACTH* adrenocorticotropic hormone, *SBP* systolic blood pressure, *DBT* diastolic blood pressure, *HR* heart rate, *Bpm* beats per minute

**Table 5:** Mixed between-within subjects’ ANOVA for pre-test, peak, and lowest value at baseline and follow-up

	*n*	Baseline to follow-up			Group			Time*group		
		*F*	*p*	Eta squared	*F*	*p*	Eta squared	*F*	*p*	Eta squared
**Pre-test**										
ACTH (pmol/L)*	85	1.63	0.205	0.02	4.51	**0.037**	0.05	0.19	0.668	0.00
Cort (nmol/L)*	82	4.27	**0.042**	0.05	0.58	0.447	0.01	0.32	0.575	0.00
SBP (mmHg)*	86	1.93	0.169	0.02	0.14	0.706	0.00	0.01	0.931	0.00
DBP (mmHg)*	86	2.49	0.119	0.03	2.66	0.107	0.03	0.89	0.349	0.01
HR (Bpm)	82	24.24	**< 0.001**	0.23	1.04	0.311	0.01	0.07	0.935	0.00
**Peak**										
ACTH (pmol/L)*	87	0.46	0.500	0.01	2.08	0.153	0.02	0.00	0.953	0.00
Cort (nmol/L)*	85	2.33	0.131	0.03	0.35	0.558	0.00	0.00	0.983	0.00
SBP (mmHg)	85	15.55	**< 0.001**	0.16	2.06	0.155	0.02	0.74	0.393	0.01
DBP (mmHg)*	85	5.14	**0.026**	0.06	5.21	**0.025**	0.06	0.59	0.444	0.01
HR (Bpm)*	84	0.09	0.763	0.00	0.20	0.657	0.00	2.47	0.120	0.03
**Lowest**										
ACTH (pmol/L)*	87	1.48	0.227	0.02	1.85	0.177	0.02	0.02	0.880	0.00
Cort (nmol/L)*	85	1.72	0.194	0.02	0.01	0.931	0.00	0.05	0.822	0.00
SBP (mmHg)*	88	0.44	0.507	0.01	0.15	0.704	0.00	0.44	0.509	0.01
DBP (mmHg)*	88	1.13	0.235	0.02	3.74	0.057	0.04	2.28	0.135	0.03
HR (Bpm)*	88	0.02	0.884	0.00	0.68	0.411	0.01	0.12	0.727	0.00

Pre-test: mean value of the − 10 and − 0 min samples taken before the test started. Peak: highest value after the test (10 or 20 min for ACTH and cortisol and during or directly after for systolic and diastolic blood pressure and heart rate). Lowest value: last value (60 min) for ACTH and cortisol, the lowest value between 10 and 60 min for systolic and diastolic blood pressure and heart rate

*ACTH* adrenocorticotrophic hormone, *SBP* systolic blood pressure, *DBT* diastolic blood pressure, *HR* heart rate, *Bpm* beats per minute

*Adjusted for sex

### Correlations between number of training sessions and response to TSST

When analyzing correlations between the number of sessions performed during the intervention and the response to the stress test, no correlation was seen for ACTH (*r* = − 0.12, *p* = 0.434), cortisol (*r* = − 0.03, *p* = 0.861), systolic blood pressure (*r* = − 0.03, *p* = 0.869), diastolic blood pressure (*r* = − 0.04, *p* = 0.818), or heart rate (*r* = − 0.07, *p* = 0.659).

## Discussion

The main result of this study is that physiological reactions to acute psychosocial stress is not significantly affected in untrained individuals performing regular exercise training for 6 months, compared to a non-training control group. Thus, neither HPA-axis responses (ACTH and cortisol) nor autonomic responses (systolic and diastolic blood pressure and heart rate) to acute psychosocial stress showed any changes following the 6-month exercise intervention. Accordingly, in this study, we could not confirm the hypothesis that aerobic exercise training will result in attenuated HPA-axis reactivity and autonomic responses to acute psychosocial laboratory stress. The participants in the intervention group significantly improved their peak oxygen uptake and time-to-exhaustion at the same time as the control group showed a reduced VO_2_ peak and time-to-exhaustion. The increase in oxygen uptake seen in the intervention group confirmed an expected effect of the aerobic exercise training performed during the intervention period. Also, the decrease in the control group confirmed compliance to the recommendation to not change their physical activity habits between baseline measures and follow-up testing.

The results showed higher pre-test values for cortisol and heart rate at follow-up irrespective of the intervention, indicating higher stress levels at arrival that might be caused by experiences from the baseline test. On the other hand, reactivity values of ACTH, cortisol and diastolic blood pressure were lower at follow-up, maybe as a result of habituation to the situation when the participants discovered that the same test was repeated. Additionally, no correlations were found between number of training sessions and reactivity to the stress test, and no differences were seen between the groups for reactivity and recovery. Thus, the result of our study does not confirm the hypothesis that regular exercise training affects HPA-axis or autonomic response to acute laboratory stress. However, the great individual variances in physiological response to acute stress and the assumed adaptation to the stress test might have affected the interpretation of the results.

There are several possible reasons for different results in the present study compared to earlier studies. Previous studies are mainly cross-sectional, comparing highly fit individuals with untrained individuals. Some of these studies showed a less pronounced physiological response for highly fit individuals [13, 18, 19, 32], while others did not [21, 33, 34]. In some cases, when studying opposites (trained/untrained), a difference can be detected. However, in our study, the change in oxygen uptake, although significant, was possibly not large enough to significantly affect the physiological responses. Maybe, the effect on the physiological stress response is only existent in highly trained individuals, which we did not study.

To the best of our knowledge, only two randomized controlled studies have been conducted measuring the physiological responses to acute psychosocial stress in relation to changes in fitness [22, 35]. Both studies showed an attenuated physiological reaction for participants allocated to exercise intervention compared to the non-exercising controls. In the study by Klaperski et al., the increase in activity level in the intervention group was comparable to our study. However, a comparison whether the increased exercise level affected fitness to the same extent as in our study is complicated due to the different methods used to measure fitness. Klaperski et al. related fitness to the participant’s relative power at individual anaerobic threshold, whereas our study used a peak oxygen uptake test. In contrast to both the study by Klaperski and co-workers and our study, the study by von Haaren et al. used an academic examination period as a real-life psychosocial stressor. The increase in VO_2_ peak was comparable to our study, but the result differed in that the intervention group in the study by von Haaren et al. showed a reduced autonomic response to the stressor. However, the types of stressors used in the studies cannot be easily compared. While our study used a 10-min laboratory, unpredictable stressor, von Haaren and co-workers used a 2-day examination period, well known to the participants. Thus, despite the similar designs in all three studies, the results diverge.

Another factor to consider when comparing studies is the labeling of exercise levels and fitness. There are large discrepancies between studies in terms of what is considered “fit” and “unfit,” both with respect to aerobic capacity and the level of exercise training. In some studies, the term “unfit” (or “low fit”) is used for individuals who do not perform any exercise [20, 32], while other studies use the same term for individuals who perform up to 4 h of exercise per week [33, 34]. At the same time, the use of the term “fit” (or “high fit”) has been used for individuals who exercise for at least 1 h per week [20], which results in an overlap of several hours of exercise per week between the terms “unfit” and “fit.”

Furthermore, the methods for determination of fitness differ between studies. Some studies used self-reported activity levels, while other studies used some kind of fitness test to evaluate the participant’s aerobic capacity. The substantial differences regarding the measures and definitions of terms related to fitness may partly explain the inconsistence of existing studies.

In our study, the participants reported themselves as untrained at screening, but this was not reflected by their level of oxygen uptake, which falls into the normal range of aerobic capacity for both men and women according to Koch et al. (2009) [36]. This result indicates that we may not have reached our intended target group of untrained individuals in the present study. Generally, when studying the effects of exercise on different physiological health measures, the greatest difference is seen between individuals with low fitness compared to individuals with a little higher fitness [37]. This might not be the case for the outcome measures of this study. Thus, the exercise training effects in the intervention group might not have been large enough to yield the physiological effects hypothesized. A screening for oxygen uptake before inclusion would have been preferable instead of relying on self-reports of physical activity level.

Another important aspect related to detecting changes on a group level is the large individual variation in physiological responses to acute stress. Thus, a larger study population is perhaps needed to detect possible effects following an exercise intervention on the physiological systems.

In this trial, women and men were analyzed together. Since we had an even distribution of women and men in both groups and adjusted for sex in the analyses, this was considered appropriate. The results showed no differences for women and men, which is in line with previous findings of Gerber et al. [19]*.*

### Strengths and limitations

The major strength of the present study is the RCT design and the relatively large study population. Including participants during the whole year diminished the seasonal effects. In addition, the personnel that performed the testing was the same during the entire time for data collection. This contributed to a familiar situation for the participants and diminished the risk of eventual intra-individual variances in the testing procedures that increase reliability of the results. Another strength is the measurement of VO_2_ peak, which present the absolute value of oxygen uptake, instead of a calculated value from submaximal assessments or self-reported levels of physical activity.

We are also aware of some limitations that need to be discussed. Firstly, as mentioned above, many of the included participants might not have been as untrained as they reported. Secondly, almost half of the participants (20 out of 47) in the intervention group had difficulty reaching the lowest acceptable level of exercise training and were thus not included in the adherence-to-protocol analyses. Changing from a low level of physical activity to engage in exercise three times per week seems to be difficult. Sherwood and Jeffery [38] reviewed factors influencing an individual’s capability to implement a physically active lifestyle. They enumerated the items identified as important and mention, for example, exercise self-efficacy, prior history of physical activity, social support, time, and access. Despite the four coaching sessions aiming to support the participants in our study, several participants found it difficult to complete the intervention. Maybe a more intensive and prolonged protocol would have resulted in a larger effect in the outcomes, but that would have required a greater achievement from both participants and research staff, which was not considered feasible in this study. Since the majority of the participants did not follow the protocol, another approach to the intervention program would have been necessary, with some supervised sessions in order to maintain the participants’ exercise level.

A third important factor to discuss is the type of stressor used in this study. Both groups showed decreased reactivity and/or reduced peak levels in all variables except heart rate in response to the TSST at the second time of the test. This indicates a habituation to the test, although previous research has suggested that when at least 4 months have passed between the tests, the risk of habituation is small [39]. In fact, a small change was made in the task at the second test, but the modification was obviously not enough for participants to experience the task as novel. The TSST may be relevant in cross-sectional studies, but in further longitudinal studies, the use of TSST in its original setting needs to be taken into consideration when the follow-up time is as short as 6 months.

The phase of menstrual cycle was not recorded which must be seen as a limitation and might have affected our results. Additionally, we had no possibilities to measure catecholamines, which would have been of interest to do, to further explore the effects on the autonomic nervous system. Lastly, it was not possible to blind the participants regarding which group they took part in, but the TSST committee was blinded and had no insight in terms of which group the participants belonged to.

## Conclusions

Regular exercise training did not affect the physiological responses to acute stress when compared to untrained controls. Thus, we cannot confirm that the cross-stressor adaptation hypothesis is a plausible mechanism explaining the stress-buffering effect of exercise training. A large-scale RCT, minimizing existing limitations of available studies, would be needed to further explore whether the cross-stressor adaptation hypothesis is valid.

## Supplementary information

**Additional file 1.** The CONSORT checklist.

## Data Availability

The dataset used and analyzed during the current study are available from the corresponding author on reasonable request.

## Abbreviations

HPA: Hypothalamic-pituitary-adrenal
ACTH: Adrenocorticotropic hormone
VO_2_ peak: Peak oxygen uptake
RCT: Randomized controlled trial
TSST: Trier Social Stress Test
AUC_i_: Area under the curve with respect to increase
ANOVA: Analysis of variance
CI: Confidence interval
